# Assessment of body-powered 3D printed partial finger prostheses: a case study

**DOI:** 10.1186/s41205-019-0044-0

**Published:** 2019-05-02

**Authors:** Keaton J. Young, James E. Pierce, Jorge M. Zuniga

**Affiliations:** 1 Department of Biomechanics, University of Nebraska at Omaha, 6001 Dodge Street Omaha, Nebraska, NE 68182 USA; 2 Facultad de Ciencias de la Salud, Universidad Autónoma de Chile, Santiago, Chile

**Keywords:** Finger amputation, 3D printing, 3D prosthetic development, 3D printed prosthetic, Additive manufacturing, Fused deposition modeling

## Abstract

**Background:**

Traditional prosthetic fabrication relies heavily on plaster casting and 3D models for the accurate production of prosthetics to allow patients to begin rehabilitation and participate in daily activities. Recent technological advancements allow for the use of 2D photographs to fabricate individualized prosthetics based on patient anthropometrics. Additive manufacturing (i.e. 3D printing) enhances the capability of prosthesis manufacturing by significantly increasing production speed and decreasing production cost. Existing literature has extensively described the validity of using computer-aided design and 3D printing for fabrication of upper limb prostheses. The present investigation provides a detailed description of the development of a patient specific body-powered 3D printed partial finger prosthesis and compares its qualitative and functional characteristics to a commercially available finger prosthesis.

**Case presentation:**

A 72-year old white male with a partial finger amputation at the proximal interphalangeal joint of the left hand performed a simple gross motor task with two partial finger prostheses and completed two self-reported surveys (QUEST & OPUS). Remote fitting of the 3D printed partial finger began after receipt of 2D photographs of the patient’s affected and non-affected limbs. Prosthetic fitting when using 3D printable materials permitted the use of thermoforming around the patient’s residual limb, allowing for a comfortable but tight-fitting socket. Results of the investigation show **i**mproved performance in the Box and Block Test when using both prostheses (22 blocks per minute) as compared to when not using a prosthesis (18 blocks per minute). Both body-powered prostheses demonstrated slightly lower task-efficiency when compared to the non-affected limb (30 blocks per minute) for the gross motor task. Results of the QUEST and OPUS describe specific aspects of both prostheses that are highly relevant to quality of life and functional performance when using partial finger prostheses.

**Conclusion:**

The use of 3D printing exhibits great potential for the fabrication of functional partial finger prostheses that improve function in amputees. In addition, 3D printing provides an alternative means for patients located in underdeveloped or low-income areas to procure a functional finger prosthesis.

## Background

Limb loss due to amputation is expected to reach nearly 3.6 million by the year 2050, which will have dramatically increased from the current 1.6 million in 2005 [1]. The majority of these amputations are considered minor amputations, as these individuals are losing only small appendages such as fingers or toes [2]. Amputation of the fingers in the upper limbs is a common occurrence and has significant implications on individuals overall function, coordination and quality of life. Loss of these appendages can reduce functional ability, resulting in difficulties performing activities of daily living (ADL) [3]. The use of prostheses has been shown to improve completion of ADLs, in addition to improving psychosocial self-esteem, body image, interlimb coordination with the contralateral limb and body symmetry [4, 5]. Despite this, prior literature found that nearly 70% of upper limb prosthetic users were unsatisfied with their prosthesis when completing ADLs [6]. In addition, it has been indicated that nearly 52% of upper limb amputees abandon their prosthetic devices due to the functional, aesthetic or other limitations [7]. In contrast to the reported figures of device abandonment, realistic rejection rates and non-usage have been estimated to be even greater due to the lack of communication between clinics and prosthetic non-users [8]. To reduce the large degree of device abandonment, it is recommended that prosthetic device fitting occur immediately or as quickly as possible following a surgical amputation, which may increase the acceptance rate of these devices [9]. Traditional prosthesis fabrication is a lengthy process that requires a certified prosthetist to make multiple castings of the affected limb using plaster, which can be both labor and material intensive. As traditional fabrication methods may not meet the rate at which prostheses must be manufactured, the need for an accelerated method of production presents itself. Modern advances in additive manufacturing (i.e., 3D Printing) have made it possible for the batch-production of low cost, customized upper-limb 3D prostheses using Fused Deposition Modeling (FDM), where the production capacity is limited to the size, type, and the total number of 3D printers available [10].

To reduce the time and inaccuracy of socket fabrication, 3D scanning has been previously utilized to scan the affected limb to allow for rapid prototyping of medical prostheses by producing accurate stereolithographic (STL) models, which are imported into computer-aided design (CAD) systems [11]. Socket fabrication using CAD methods have been shown to be reliable when coupled with digital files (i.e. STL’s) and additive manufacturing (i.e. FDM) reducing the amount of time needed to fabricate prosthetic sockets [12]. Furthermore, CAD systems have been shown to be a viable alternative for fabrication of functional 3D printable transitional prostheses with highly customized sockets relative to patient-specific anthropometrics [13]. Transitional prostheses are referred to as “temporary prosthesis” or “immediate postoperative prosthesis”, and have been previously investigated for retention and restoration of muscular strength and range of motion [14]. Therefore, the purpose of the present study is twofold: (i) to describe the development of a transitional 3D printed prosthesis for partial finger amputees and (ii) to discuss the qualitative and functional characteristics when compared to a commercially available partial finger prosthesis. This information is essential in creating a compelling argument for the efficacy of using 3D printed prostheses as transitional prosthetics for amputees with partial finger loss. We hypothesize that the locally fabricated 3D printed prosthesis will produce similar qualitative and functional results when compared to the commercially available partial finger prosthesis. Our hypothesis is based on previous investigations that have shown the use of 3D printing for the development of functional prostheses [13].

## Case presentation

### Research subject

**Fig. 1:**
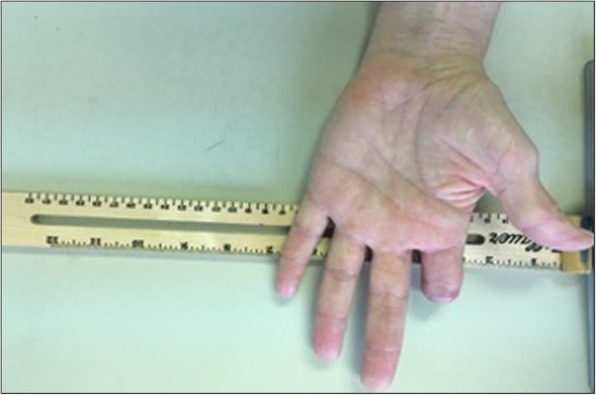
Research subject with amputation at the proximal interphalangeal joint of the left hand

The subject evaluated in this study was a 72-year-old male (height 177.8 cm; weight 81.6 kg) with an acquired traumatic amputation of the index finger at the proximal interphalangeal joint (PIP) on the left hand (non-dominant) distal to the metacarpal joint (MCP) (Fig. 1). The residual limb of the affected hand was 4.5 cm in length (MCP to PIP) and 7 cm in circumference. The non-affected finger on the right hand (dominant) was 9.5 cm in length and 7 cm in circumference. Prior to the laboratory visit, this participant provided pictures of both the affected and non-affected hands for the remote fitting of the 3D printed finger prosthesis [10]. The subject had acquired the MCP-Driver™ finger prosthesis (NAKED Prosthetics Inc., Olympia, WA USA) and reported that they regularly used the device prior to participating in this study. The local Institutional Review Board approved this study.

### Local 3D printed finger prosthesis

**Fig. 2:**
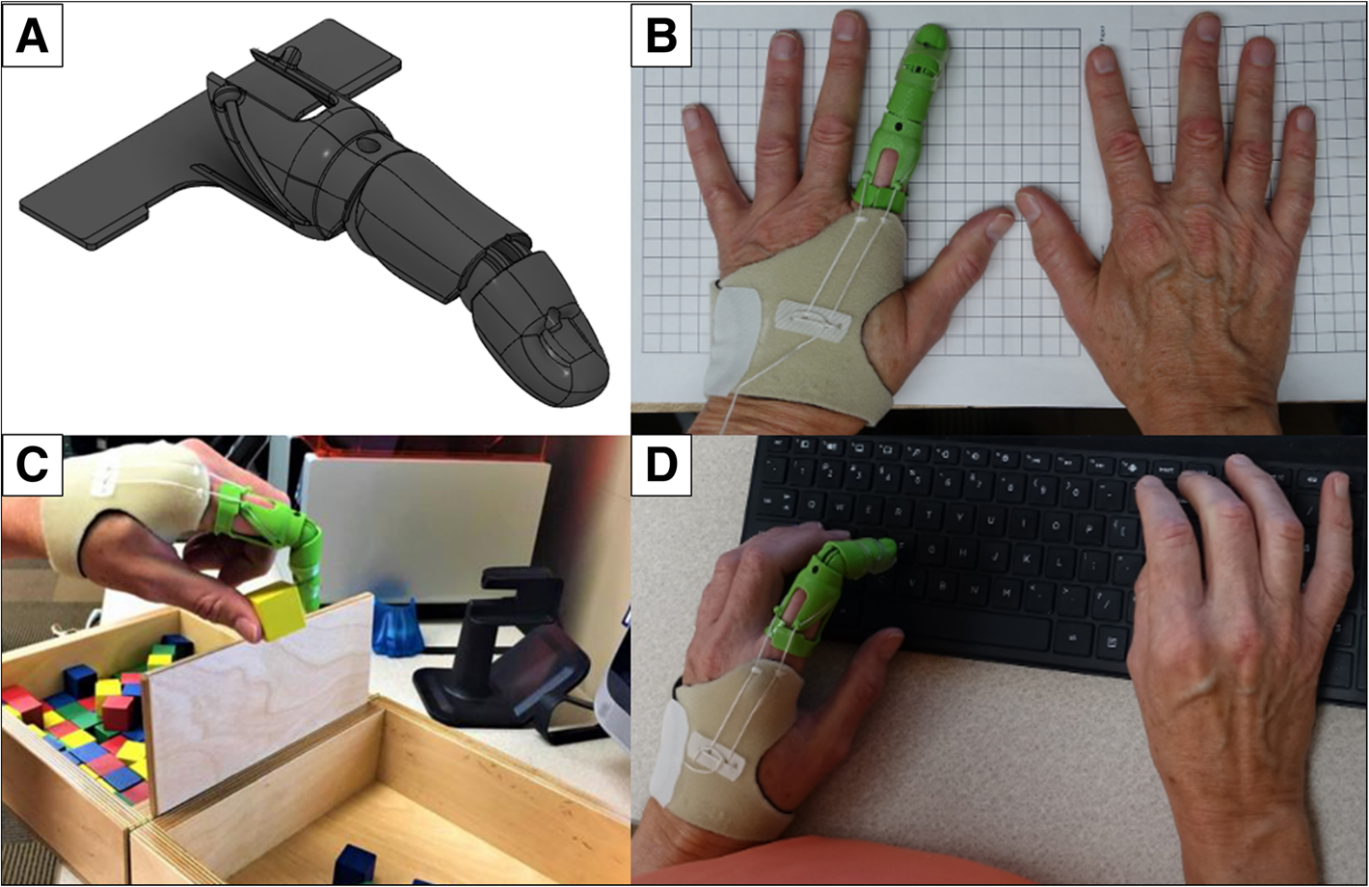
**a** Rendered CAD model of LFP. **b** Hand symmetry between the non-affected hand and affected hand with 3D printed finger prosthesis. **c** Participant performing the Box and Block Test. **d** Participant typing on an electronic keyboard

The locally 3D printed finger prosthesis (LPF) was created by the authors for the purpose of testing a prototype of a body-powered partial finger prosthesis. The featured design utilizes a tension-driven voluntary-closing (VC) mechanism, which requires activation of the residual limb’s musculature at the MCP to produce flexion for coordinated manipulation of objects. The prosthesis was designed utilizing the participant’s non-affected finger length, width and circumference to create an approximately sized prosthetic limb to match the non-affected limb. The LPF allows for pinch grasping actions actuated by flexion of the MCP. An MCP flexion angle of 40° produced 1 in. of cable travel for full operation. Drafting and design of this device utilized multiple different methods ranging from parametric design to model sculpting when using a computer-aided design program (CAD) (Autodesk Fusion 360, Autodesk Inc., San Rafael, CA USA). Overall design of the LFP incorporates three segmented sections with simple pivot joints in-between each segment to provide smooth and coupled movement. These sections include the proximal, middle and distal segments. Joints between the middle and proximal segment allow for movement, however, the joint between the distal and middle segments is frozen with the distal segment of the finger flexed at a 30° angle downward to allow for smoother actuation of the finger when tension is applied by the user. A rear flat portion is sized to the circumference of the subject’s finger and then thermoformed around the finger during fitting and acts as a retention ring to provide additional stability to the finger. The device was secured using a customized soft neoprene cast fitted to the palm of the hand, which was used to create an anchoring point for actuation of the device to occur and to reduce the amount of friction that the user may experience from the nylon string utilized to produced rotational force around the LFP’s interphalangeal pivot joints. A silicone grip is added onto the fingertip to increase grasp compliance and to prevent slippage of gripped objects. Initial sizing of the prosthesis was performed remotely and began by instructing the patient to photograph both the affected and unaffected limbs including a known measurable scale, such as metric grid paper. This photogrammetric method allowed the extraction of several anthropometric measurements from the photographs, including limb length, width and circumference derived from the limb area. This photograph was then uploaded to a CAD software, which was calibrated by the known unit of measure of the graph paper (i.e. 1 cm boxes) included in the photograph (Fig. 2). Patient anthropometric measures including limb length, width and circumference were utilized to create a socket that was then incorporated into the full design. Once all measure had been validated by a certified prosthetist, STL model files were uploaded to a 3D slicer software (Simplify3D v4.1, Simplify3D, Blue Ashe, OH) to add any material support that would be needed during the printing process. Sliced 3D files were then transferred to a desktop 3D Printer (Ultimaker 2 extended, Ultimaker B.V., Geldermalsen, The Netherlands). The material used in printing the finger prostheses was polylactic acid (PLA). The prosthesis was printed at 35% infill using a rectangular infill pattern, 60 mm/s print speed, 150 mm/s travel speed, 210 °C nozzle temperature, 50 °C heated build plate, 0.15 mm layer height, and 0.8 mm shell thickness. The design of the LFP allows for all motion components to be printed in place and pre-assembled. Additional components of the finger prosthesis include Nylon string (1 mm diameter) and elastic cord (1.5 mm diameter), which produce the flexion and extension capabilities observed in the functionality of the device. Additional components include medical-grade padded foam as a soft socket and anchoring point and a protective skin sock for the residual limb to reduce friction of the prosthesis on the skin.

**Fig. 3:**
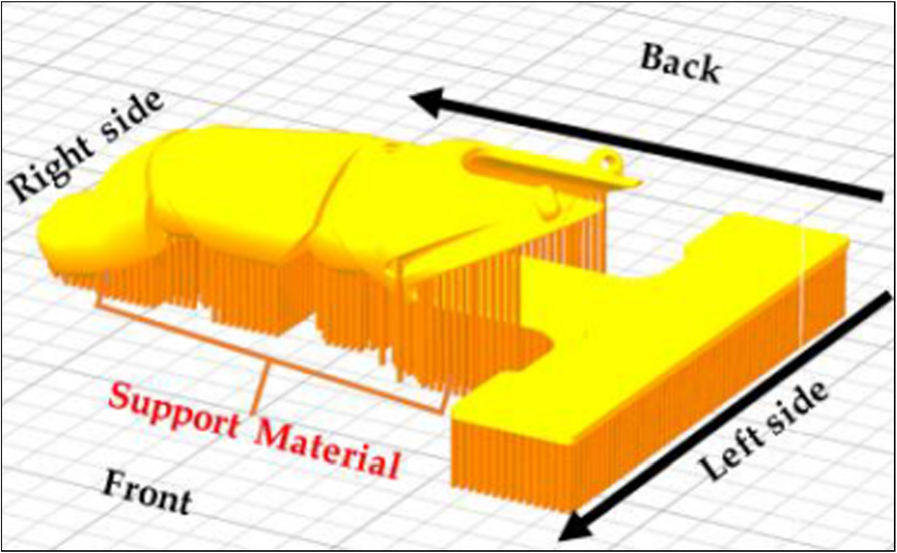
Build orientation and support generation of the 3D printed finger prosthesis

The LFP was manufactured using PLACTIVE™ (PLACTIVE™ 1% Antibacterial Nanoparticles additive, Copper3D, Santiago, Chile), which is formulated with an internationally patented additive containing copper nanoparticles. Copper nanoparticles have been shown to be effective in eliminating fungi, viruses, and bacteria, but are harmless to humans [15]. PLACTIVE™ was chosen as it uses a sound and proven antibacterial mechanism, is a low-cost material that is biodegradable, and possesses thermoforming characteristics that facilitate post-processing and final adjustments of the LFP. PLACTIVE™ has similar physical (relative viscosity = 4.0 g/dL, clarity = transparent, peak melt temperature = 145–160 °C, glass transition temperature = 55–60 °C) and mechanical (tensile yield strength = 8700 psi, tensile strength at break = 7700 psi, tensile modulus = 524,000 psi, tensile elongation = 6%, flexural strength = 12,000 psi, and heat distortion temperature at 66 psi = 55 °C) properties to standard PLA. The average printing time for the LFP was 60 ± 5.6 min. Post-processing consisted of support removal and filing of rough areas in the joints and prosthetic socket area in contact with the skin. The build orientation on the build platform and generation of the support are illustrated in Fig. 3. Support was generated for all overhang angles of 45° or higher required support material. The post-processing of the LFP took 10 min, and assembly took 30 min. The total material cost of the LFP was estimated to be $20, due to the multiple prototypes made throughout the development process.

### Commercial finger prosthesis

**Fig. 4:**
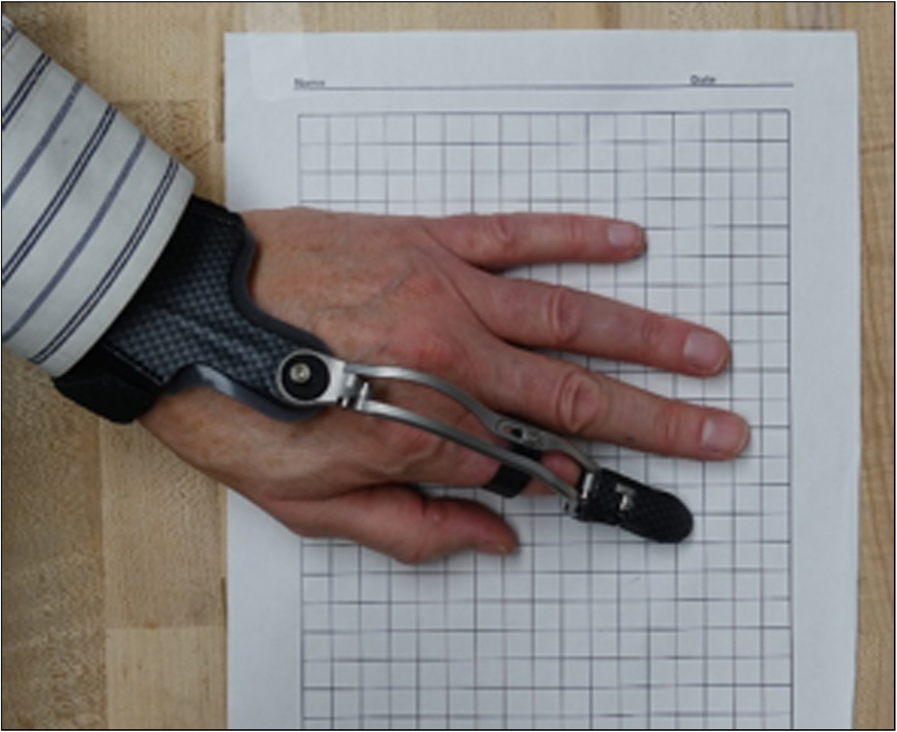
NAKED Prosthetics Inc. MCP-Driver Finger Prosthesis

The commercial prosthesis investigated in this study is the MCP-Driver*™* (MFP) and is manufactured by NAKED Prosthetics Incorporated. This partial finger prosthesis is body-powered and utilizes a linkage-driven mechanism of action for the articulation of a partial finger amputation. Overall device design specifically accommodates patients that have acquired a proximal phalanx amputation and aims to restore articulation at the middle and distal phalanges. The MFP allows for specific grasping orientations such as pinching, key, and cylindrical types and provides good grip stability. Capabilities of this device include modular force production, as the user is able to modify the amount of force that is directed onto the object that is being grasped allowing for more sensitive objects to be held (i.e., Egg). The MFP is fabricated using titanium, 316 stainless steel, silicone and medical grade nylon, enhancing durability and aesthetic appearance (Fig. 4). The estimated time to fabricate one of these devices is between 6 to 8 weeks, which allows for the collection of proper documentation, photos, and casts from prosthetic practitioners to be received. Multiple MFP devices can be used in the case of multiple amputations at the proximal phalanx, which are anchored to a carbon fiber cast around the palm of the affected hand. The ability to attach multiple prostheses to a centralized anchor-point allows for improved intralimb coordination, force production and overall function of the affected hand. The MFP has the ability for finger adduction and abduction due to articulation at the anchoring point of the cast and can be adjusted by the clinician providing care or the user. Additionally, by increasing the planes of motion that the prosthetic device can move within, the acclimation period to the prosthetic device can be significantly reduced. An adjustable carbon fiber ring is used as a minimal socket to allow for the suspension of the finger. The tensile strength of this ring is 80 lbs. In addition, this simplified socket design improves overall comfort and reduces infringement of the device on range of motion of the fingers [16]. The MFP is available in multiple different aesthetic coatings, which can be selected at the discretion of the user. The MFP is fabricated using a combination of metal 3D printing technology and externally applied components that allow for manual adjustment of the device for a more comfortable fit, which contrasts to the LFP that uses FDM printing and thermoplastics. The cost of this device is estimated to be $9000 to $19,000 per device and highly depends on the parts used for fabrication of the device. In order to obtain accurate information pertaining to this specific device, the authors privately communicated with the manufacturer for more detailed information of specific device discussed in this investigation.

### Procedure

**Fig. 5:**
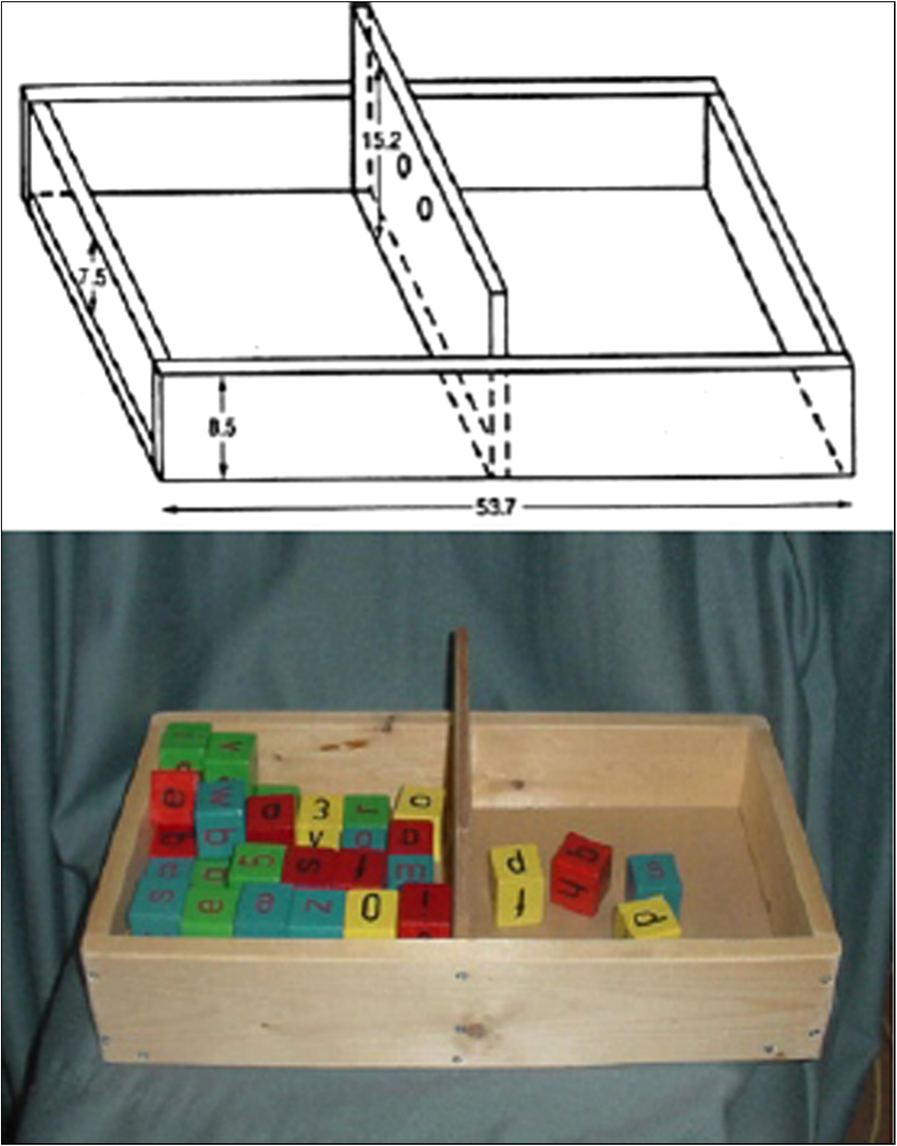
Box and Block Test (BBT) and structural dimensions (cm)

The research subject visited the research laboratory twice, during the first visit an orientation session took place where the testing procedures were fully explained, informed consent was completed and the initial device distributed for use. During the second visit, the subject was given a version of the initial device that was modified for improved comfort, and testing procedures were completed. The Box and Block Test (BBT) was completed, which acts as a functional outcome measure of unilateral gross manual dexterity. The BBT has been used in previous prosthetic assessment studies and has been seen to measure prosthetic limb performance and motor learning [17, 18]. The Box and Block test required the subject to move 1-in. blocks one at a time from one box, over a partition, and to drop the blocks in the adjacent box (Fig. 5). The subject was seated comfortably, and then completed a one-minute trial of the BBT with his affected hand, unaffected hand, and affected hand with the 3D printed prosthesis and MFP The subject was asked to place their hands on the sides of the box. As testing started, the subject was asked to grasp one block at a time, transport the block over the partition, and release it into the opposite compartment.

Two weeks after laboratory testing concluded, the subject was sent a satisfaction survey that utilized two separate prosthetic specific surveys. Prosthesis use and satisfaction were assessed using the Quebec User Evaluation of Satisfaction with assistive Technology (QUEST 2.0) [19]. The QUEST 2.0 consists of 12 items with optional satisfaction items that a participant may feel are essential to a particular aspect of their device or service. All responses were graded on a five-point satisfaction scale ranging from one (“not satisfied at all”) to five (“very satisfied”). The QUEST 2.0 utilizes three separate categorization scores including: Device, Service, and average total score of all 12 items based on the range of 1 to 5. The “service” portion did not pertain to this study and therefore the “device” portion of the QUEST was the only one assessed, which encompassed only 8 items. In addition, the Orthotics and Prosthetics Users Survey (OPUS) was used to evaluate the subjects upper extremity functional status and quality of life [20]. The OPUS consists of five subscales; of these, the upper extremity functional status (UEFS) and OPUS-Satisfaction with Devices (CSD) were used, which both use a qualitative rating scale of “Strongly Agree” to “Don’t Know/Not Applicable”. The upper extremity functional status survey consists of 28 questions pertaining to the completion of physical tasks (i.e., Use key in lock, Put on socks). The OPUS – CSD consists of 21 questions that observe the opinion of the user concerning the overall device function and services provided for the device (Table 1). The first 11 questions of the OPUS – CSD were utilized in this study, as the other items lacked in relevance.

**Table 1:** Orthotics and Prosthetics User Survey - Satisfaction with Devices (OPUS - CSD)

Please mark the response that most closely reflects your opinion:	Local Prosthetic	Commercial Prosthetic
My prosthesis fits well.	Agree	Agree
The weight of my prosthesis is manageable.	Agree	Agree
My prosthesis is comfortable throughout the day.	Agree	Agree
It is easy to put on my prosthesis.	Agree	Agree
My prosthesis looks good.	Agree	Agree
My prosthesis is durable.	Disagree	Agree
My clothes are free of wear and tear from my prosthesis.	Strongly Agree	Agree
My skin is free of abrasions and irritations.	Agree	Agree
My prosthesis is pain free to wear.	Don’t Know/ Not Applicable	Agree
I can afford the out-of-pocket expenses to purchase and maintain my prosthesis.	Agree	Don’t Know/ Not Applicable
I can afford to repair or replace my prosthesis as soon as needed.	Agree	Don’t Know/ Not Applicable

*Response Range:* Strongly Agree, Agree, Neither Agree nor Disagree, Disagree, Strongly Disagree, Don’t Know/ Not Applicable

## Results

In the performance of the gross manual dexterity task, the subject moved 18 blocks per minute (BPM) when no prosthesis was utilized. When a prosthesis was used, performance improved to 22 BPM during the one-minute trial with both the LFP and the MFP. Comparatively, the non-affected limb moved 30 BPM, which demonstrates the relative functional difference of the affected limb.

Results of the QUEST 2.0 showed that the LFP scored slightly higher (3.3 ± 1.2), as compared to the MFP (2.5 ± 0.5) device satisfaction. Description of the results for the QUEST 2.0 survey for both the LFP and the MFP are observed (Table 2). Qualitative results for the OPUS – CSD are displayed in Table 1. As only 3 of the 28 questions were completed for the OPUS – UEFS, only completed questions for both prostheses are discussed. The OPUS – UEFS indicated that the participant was able to complete the tasks: “Carry a laundry basket” and “Put on and take off prosthesis” very easily with both prostheses and “Open a bag of chips with both hands” easily with the LFP and very easily with the MFP.

**Table 2:** Quebec User Evaluation of Satisfaction with assistive Technology (QUEST) Ratings

How Satisfied Are You With:	Local Prosthetic	Commercial Prosthetic
Dimensions (size, height, length, width)	4	3
Weight	4	3
Adjustments (fixing, fastening)	2	2
Safety (secure)	2	2
Durability (endurance, resistance to wear)	4	2
Ease of Use	5	2
Comfort	3	3
Effectiveness (degree to which device meets your needs)	2	3
Device Satisfaction	**3.3 ± 1.2**	**2.5 ± 0.5**

1 = not satisfied at all, 2 = not very satisfied, 3 = more or less satisfied, 4 = quite satisfied, 5 = very satisfied. Total Device, Service and Satisfaction means calculated using average of each item

## Discussion

The primary findings of the current investigation provided evidence that the LFP produced very similar functional results to that of the MFP (Table 3). The results from the current investigation provides evidence demonstrating that both a tension- and linkage- driven body-powered prostheses can produce similar performance outcomes in the BBT. The subject demonstrated a lower functional outcome in the BBT when not using a prosthesis on the affected hand, with 60% task-specific efficiency when compared to the non-affected hand. The use of prostheses improved the task-specific efficiency to 73% of the non-affected hand, suggesting that the use of either the LFP or MFP will improve gross dexterity.

**Table 3:** Box and Block Test

Condition	Blocks per Minute
Local Prosthetic	22
Commercial Prosthetic	22
No Prosthesis	18
Non-Affected Hand	30

The current investigation examined the subject’s experienced satisfaction when using both of the prostheses. From the QUEST 2.0 device satisfaction survey, it was observed that the LFP received a slightly higher satisfaction rating, with the most critical satisfaction items being Comfort, Effectiveness, and Adjustments for the LFP, and Comfort, Effectiveness and Weight for the MFP. To supplement the QUEST 2.0 results, the OPUS – CSD provides a qualitative description of the subject’s feelings toward specific aspects of the prosthesis used. The questions used in the OPUS – CSD are similar to those of the QUEST, thus helping to provide more insight into the overall satisfaction with these devices. It can be observed from the OPUS – CSD that the MFP shows no faults in functional or aesthetic aspects, however, may not offer the same affordability or replacement capability as compared to the LFP. In addition, the LFP met the durability standard of the subject in the QUEST but did not meet the standard in the OPUS – CSD; this may indicate such confounding factors as varied survey times, which could lead to a difference in opinion between devices. Lastly, it can be seen that overall comfort was the same across both prostheses, which is important when considering the potentially abrasive surface finish of the additively manufactured parts.

From the OPUS – Upper Extremity Functional Status survey it was observed that only 3 of the 28 questions were answered fully, making qualitative comparisons between these prostheses difficult for the current participant. This substantial difference in response type (i.e. Not Applicable or Very Easy) may be due to the limited amount of time that the subject was given to use the LFP. The MFP was shown to have significantly more use due to the substantially higher number of question completion, as compared to the LFP. The vast majority of all tasks were observed to be relative “Easy” or “Very Easy”, with the exceptions of “Twisting a lid off a small bottle” and “peeling potatoes with a knife/peeler”. From the UEFS portion of the OPUS, it can be seen that the MFP is very functional and easy to use and can be used for a broad range of ADLs. In general, each prosthetic device provides different functional implications that individuals may find valuable throughout specific activities in daily life. As adequate qualitative measures could not be obtained for both devices in the current investigation, any implications derived from the qualitative results should be considered finite. Based on the information provided, neither prosthetic can be advocated for over the other; therefore, further information should be collected concerning device satisfaction.

Limitations of the present investigation are related to the number of trials performed during functional testing, a limited number of materials used in fabrication, use of only one testing protocol, and the amount of time the subject used one device compared to another. Specific limitations of the LFP relate to the limited functionality and fitting of the device, as it only provides the ability to flex and extend the artificial appendage, compared to the multiple planes of movement allowed by the commercially available MFP. In addition, the must be thermoformed to the individual’s affected limb, which requires experience fitting a prosthetic device and an understanding of thermoplastic element of the filament used in the fabrication process. Furthermore, a more comprehensive testing protocol should be used when evaluating the overall functionality of a prosthetic device to include benchmark factors such as friction coefficient, compliancy, and cycling tests to ensure the reliability of a specific device over time. As the survey results showed some valuable information, many of the questions were unable to be answered due to inadequate time with a specific prosthesis and therefore a longer period of use should be allowed prior to survey administration.

Future prototypes of 3D printed finger prostheses may encompass different mechanisms of action such as myoelectric, tension-, or linkage- driven mechanisms. Future studies should test a larger sample size using different prostheses, each with its own unique mechanism of action. While the primary findings of this case study indicate that a LFP can compare to the functional improvement seen in commercially fabricated prosthesis, the overall quality of life outcomes are not as definitive. It is clear that using a finger prosthesis for amputation at this specific amputation level are beneficial, however, further investigations must be performed to validate the efficacy of using these prostheses for amputations that are either more or less significant than the case displayed in this investigation.

## Conclusion

The current investigation described two different types of body-powered finger prostheses and observed the functional and satisfaction outcomes of a single subject. An improvement in function is evident when either prostheses was used, with principal differences between the prostheses being the method of fabrication, design, and overall mechanisms of action. As the accessibility to 3D printing continues to enlarge, there is great potential for 3D printing to pave the way for multiple new medical applications and devices, which may transform the fabrication process of future medical devices.

## Abbreviations

ADL: Activities of Daily Living
BBT: Box and Block Test
BPM: Blocks per Minute
CAD: Computer-Aided Design
CSD: Satisfaction with Devices
FDM: Fused Deposition Modeling
LFP: Local 3D Printed Finger Prosthesis
MCP: Metacarpal Joint
MFP: MCP-Driver*™*
OPUS: Orthotics and Prosthetics Users Survey
PIP: Proximal Interphalangeal Joint
PLA: Polylactic Acid
QUEST: Quebec User Evaluation of Satisfaction with Assistive Technology
STL: Stereolithographic
UEFS: Upper Extremity Functional Status
